# Rationally designed bimetallic Au@Pt nanoparticles for glucose oxidation

**DOI:** 10.1038/s41598-018-36759-5

**Published:** 2019-01-29

**Authors:** Kyubin Shim, Won-Chul Lee, Yoon-Uk Heo, Mohammed Shahabuddin, Min-Sik Park, Md Shahriar A. Hossain, Jung Ho Kim

**Affiliations:** 1 Institute for Superconducting and Electronic Materials (ISEM), Australian Institute for Innovative Materials (AIIM), University of Wollongong, North Wollongong, NSW 2500 Australia; 2 Institute of BioPhysio Sensor Technology (IBST), Pusan National University, Busan, 46241 Republic of Korea; 3 Graduate Institute of Ferrous Technology (GIFT), Pohang University of Science and Technology (POSTECH), San 31, Hyoja-Dong, Pohang, 37673 Republic of Korea; 4 Department of Physics and Astronomy, College of Science, King Saud University, P.O. Box 2455, Riyadh, 11451 Saudi Arabia; 5 Department of Advanced Materials Engineering for Information and Electronics, Kyung Hee University, 1732 Deogyeong-daero, Giheung-gu, Yongin-si, Gyeonggi-do, 17104 Republic of Korea; 6 School of Mechanical & Mining Engineering, The University of Queensland, Brisbane, QLD 4072 Australia

## Abstract

Bimetallic nanoparticles (NPs) have aroused interest in various fields because of their synergetic and unique properties. Among those nanoparticles, we strategically approached and synthesized Au@Pt NPs *via* the sonochemical method with different molar ratios (e.g. 3:7, 5:5, and 7:3) of Au to Pt precursors. The particle structure was confirmed to be core-shell, and the size was estimated to be 60, 52, and 47 nm, respectively, for 3:7, 5:5, and 7:3 ratios of Au to Pt. The detailed structure and crystallinity of as-prepared Au@Pt NPs were further studied by scanning electron microscopy, transmission electron microscopy with element mapping, and X-ray diffraction. It should be noted that thickness of the dendritic Pt shell in the core-shell structure can be easily tuned by controlling the molar ratio of Au to Pt. To explore the possibility of this material as glucose sensor, we confirmed the detection of glucose using amperometry. Two dynamic ranges in a calibration plot were displayed at 0.5–50.0 µM and 0.05–10.0 mM, and their detection limit as glucose sensor was determined to be 319.8 (±5.4) nM.

## Introduction

Metal nanoparticles (NPs) have attracted significant attention in various fields because of their unique chemical and physical properties, which can be easily tuned by tailoring their size, shape, and composition. Surface area and crystallinity also affect their properties^1^. Among these NPs, noble metals, including Pt, Au, Ag, and Pd, are particularly important for catalysts^2,3^, sensors^4,5^, photonics^6,7^, and medicine^8,9^. As explained in one of the most extensive studies, Pt NPs are highly attractive due to their superior catalytic activity, despite their high cost^2^. To overcome this issue, various mesoporous architectures have been employed to reduce the relative Pt content. In particular, the catalytic efficiency can be easily improved through a large surface-to-volume ratio. In addition to these methods, composition changes to partially replace the Pt by the addition of carbon and/or metals (Fe, Co, Ni, Pd, Au, and Ag) have also been accelerated for greater catalytic activity and durability^10–18^.

According to this research background, bimetallic NPs are expected to exhibit unique properties, not only a combination of the properties characteristic of the two metals, but also due to the synergetic effects of the two metals^19^. It is well known that bimetallic NPs are synthesised by various methods: chemical reduction, thermal decomposition, biosynthesis, galvanic replacement, and sonochemical and radiolytic methods^10^. The structures of bimetallic NPs are classified into three types: (i) core-shell, (ii) cluster in cluster, and (iii) random alloy and alloy structures. The shape and size of bimetallic NPs are precisely determined by their preparation methods and conditions. Due to these properties, bimetallic based NPs, which have the structures of nanospheres^20,21^, nanowires/tubes^22,23^, nanosheets^24,25^, and nanocages/frames^2,26^, have been suggested and employed for different catalysis and sensor applications.

In detail, a range of bimetallic based NPs are used as electrode materials in the sensing field to detect methanol^27^, glucose^28^, phenol^29^, hydrogen peroxide^30^, and biologically important small organic molecules^31^. Among them, glucose oxidation reactions in particular were included in our study because diabetes mellitus has contributed greatly to human suffering. Many electrode materials have already been reported for non-enzymatic detection of biologically important organic compounds, including glucose. It is still necessary, however, to find new catalytic electrode materials to enhance sensing performance in neutral solution. Metal based electrode materials are known to show the good catalytic performance in basic solution. In neutral solution, however, glucose oxidation is even more important for practical industry and biological application fields. Hence, composites of Au and Pt NPs are still being explored as potential candidate electrode materials. Therefore, we selected Au and Pt composite material to form core-shell structure for a highly sensitive glucose oxidation reaction.

In this work, we synthesised core-shell Au@Pt structures with different molar ratios (Au^3+^ to Pt^2+^ (denoted as Au@Pt) = 3:7, 5:5, 7:3) by a surfactant-assisted process using the ultrasonic irradiation method^32^. Changing the precursor’s content ratio is expected to modify the thickness of the Pt shell in the core-shell structures of Au@Pt NPs. The NPs were precisely characterized by transmission electron microscopy (TEM), wide-angle powder X-ray diffraction (XRD), cyclic voltammetry (CV), and chronoamperometry (CA). We finally examined the glucose oxidation reaction according to the thickness of the Pt shell, which resulted from the different composition ratios of Au to Pt.

## Results and Discussions

**Figure 1:**
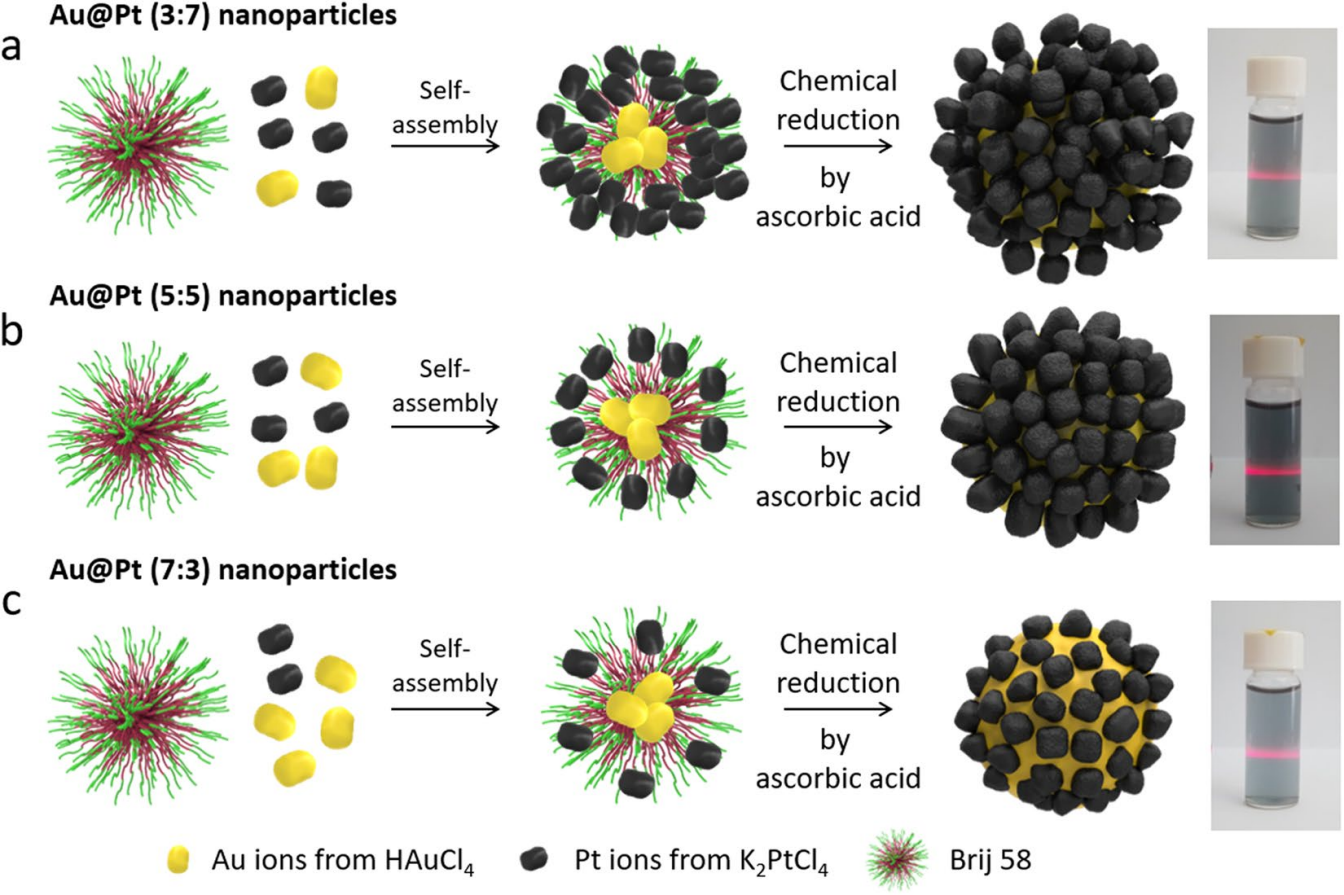
Illustrations of synthesis of Au@Pt nanoparticles with three different Au:Pt ratios (**a**) 3:7, (**b**) 5:5, and (**c**) 7:3 (left), and photographs of the Tyndall effect on Au@Pt suspensions in solution (right).

For the synthesis of core-shell structured Au@Pt nanoparticles (NPs), amphiphilic HAuCl_4_ and K_2_PtCl_4_ in different molar ratios (Au:Pt = 3:7, 5:5, 7:3) with Brij 58^®^ (Brij 58) were reacted using the sonochemical method (Fig. 1). Firstly, the Au (20 mM) and Pt (20 mM) precursor solutions were mixed in different molar ratios, and then Brij 58 solution was added into each solution. Secondly, ascorbic acid (AA) solution as a reducing agent was mixed with each prepared solution under ultrasonication. The procedure involved the use of the reducing agent AA to obtain the core-shell structured Au@Pt from the two precursors (K_2_PtCl_4_ and HAuCl_4_). Finally, the prepared products were washed several times with DI water and ethanol to remove the surfactant and reactants. We also confirmed the presence of nanoparticles in solution by the Tyndall effect (right photographs of Fig. 1). We argue that Pt and Au have different reduction kinetics and potentials^32^, which result in the core-shell structure of the Au@Pt.

**Figure 2:**
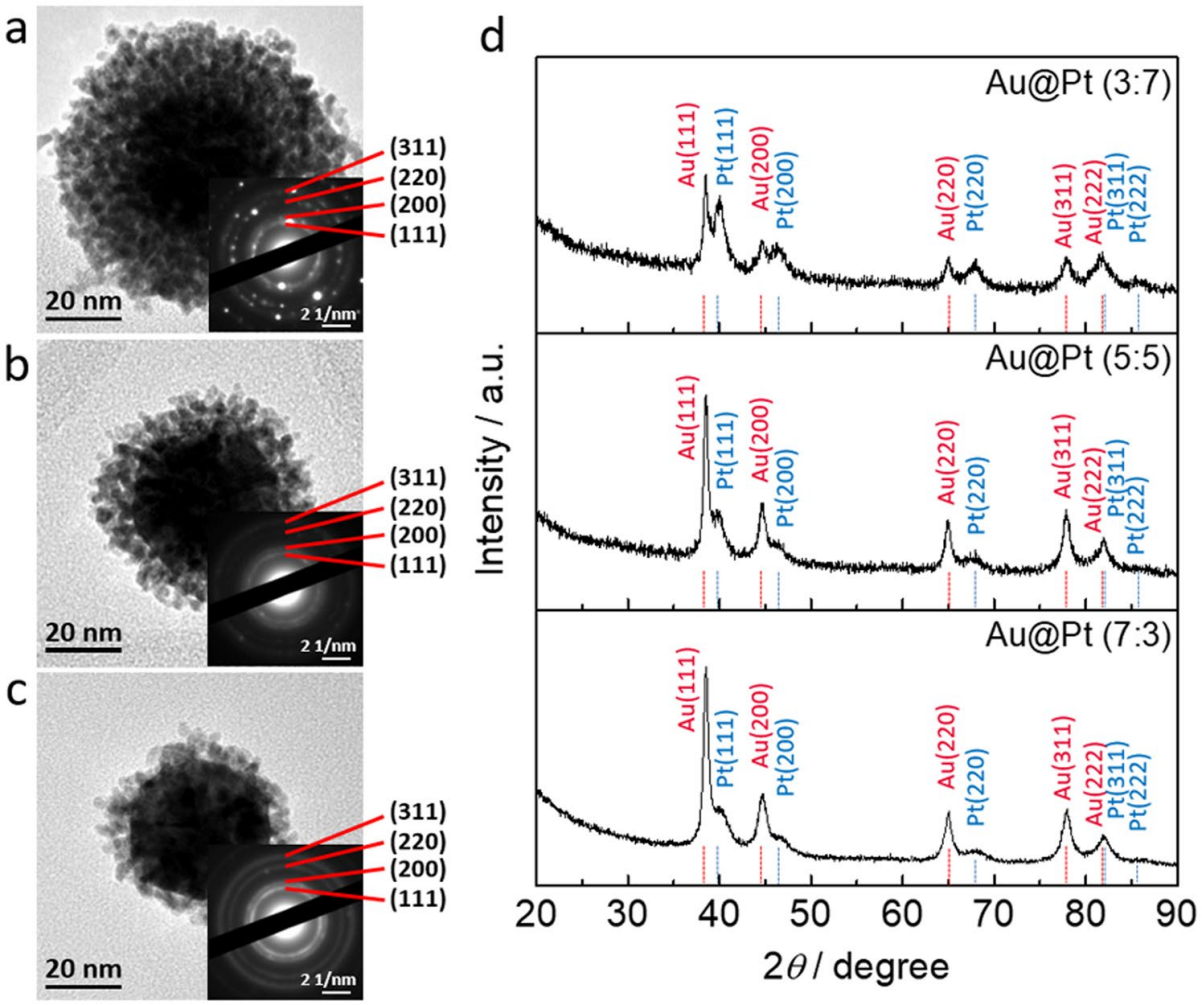
(**a**–**c**) TEM images of Au@Pt NPs with three different Au:Pt ratios (**a**) 3:7, (**b**) 5:5, and (**c**) 7:3, with the insets showing the corresponding SAED patterns. (**d**) Wide-angle XRD patterns of Au@Pt NPs with three different ratios (3:7, 5:5, 7:3).

Different composite ratios of Au@Pt NPs were obtained at room temperature, and the formation of NPs with different sizes and shapes was confirmed using TEM (Fig. 2 and S1). The average diameters of the NPs obtained from different molar ratios of Au to Pt (3:7, 5:5, and 7:3) were estimated to be around 60, 52, and 47 nm, respectively, as can be seen in Figs 2a–c and S1. With increasing the Au ratio, the entire diameter gradually decreased. To further confirm the trend of size following by different Au:Pt ratios, particle size distribution of Au@Pt NPs with different Au:Pt ratios (3:7, 5:5, and 7:3) were obtained by dynamic light scattering (DLS), respectively (Fig. S2). As the ratio of gold increased, it was confirmed once again that the size became smaller. In addition, SEM images showed that the Au@Pt NPs have the uniform size (Fig. S3). In Fig. 2a–c, we selected one particle which has the same size of Au core for comparing the thickness of Pt shells. The Au core shows negligible change, indicating that the Pt dendritic structures strongly affect the diameter of the core-shell structure. The selected area electron diffraction (SAED) patterns of the three kinds of Au@Pt NPs indicate that they have faced centred cubic (*fcc*) crystal structures (Fig. 2a–c insets). Figure 2d shows the wide-angle XRD patterns for each ratio of Au@Pt NPs. The patterns for the Au@Pt NPs also indicate the *fcc* structure, which corresponds to several peaks from the (111), (200), (220), (311), and (222) planes. When the relative amount of Au increases, the Au peak intensity proportionally increases, which is obvious evidence that the ratios of the different types of Au@Pt NPs are different.

**Figure 3:**
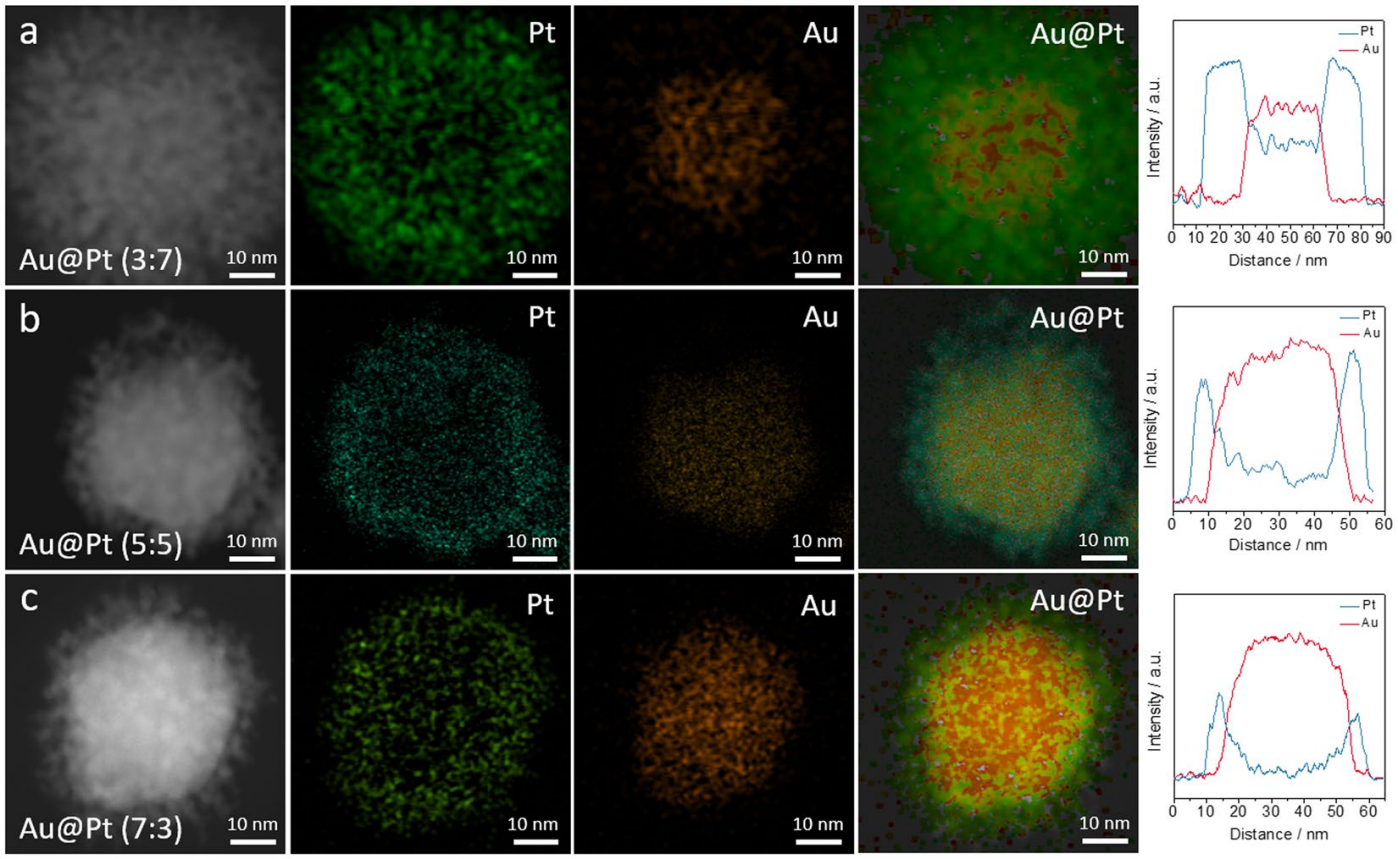
High-angle annular dark-field scanning transmission electron microscope (HAADF-STEM) images, corresponding elemental mappings, and line scans of Au@Pt NPs with different molar ratios: (**a**) 3:7, (**b**) 5:5, and (**c**) 7:3.

To further understand the distribution of Au to Pt, element mapping and line scans were performed on the Au@Pt NPs with the different molar ratios (Fig. 3). We observed that Au is located in the centre (core) of the NPs, while Pt is located on the outer edge of the Au core (shell) in the form of dendritic arms. As the above TEM images showed, the particles ranged from 60 to 47 nm in size (Fig. S1). In particular, the Au core size is estimated to be from 35 to 50 nm, and the thicknesses of the Pt shell are from 5 to 20 nm. Herein, the low intensity of the Au core in Au:Pt (3:7) is due to the greater thickness of the Pt dendritic shell. These results were also confirmed by line scanning of Au@Pt NPs. We confirmed that, as the content of Pt was decreased, the thickness of the Pt shell also was decreased.

**Figure 4:**
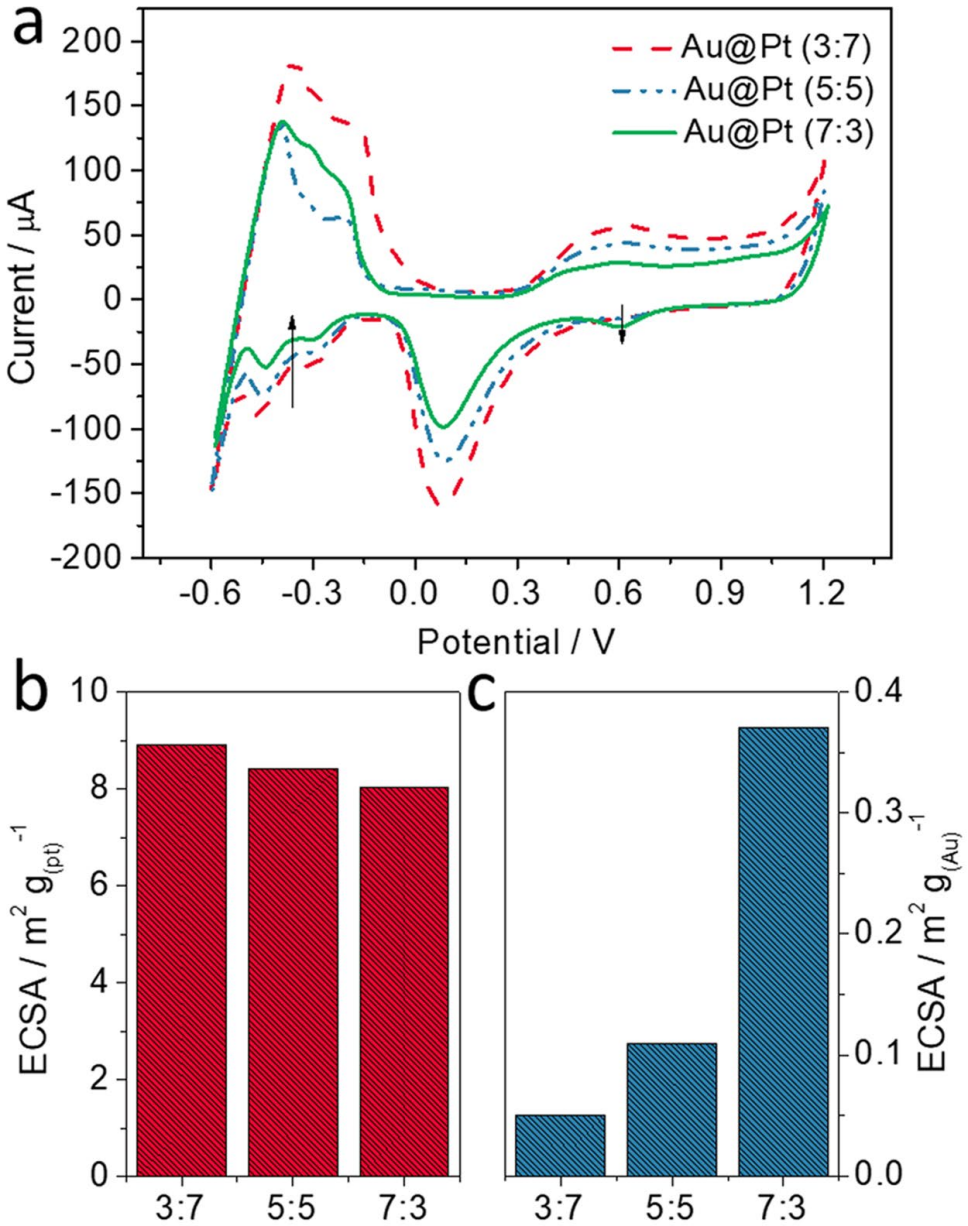
CVs and ECSAs for the three different ratios (3:7, 5:5, 7:3) of Au@Pt NPs. (**a**) CVs in 0.5 M H_2_SO_4_ (scan rate:100 mV s^−1^), (**b**) ECSA from adsorption of H atoms, (**c**) ECSA from cathodic peaks of Au.

To calculate the electrochemically active surface areas (ECSA), cyclic voltammograms (CVs) were recorded for Au@Pt (3:7, red, dashed), Au@Pt (5:5, blue, dashed), and Au@Pt (7:3, green, solid) NPs (Fig. 4a). The CVs were recorded in 0.5 M sulfuric acid (H_2_SO_4_) solution from −0.6 V to +1.2 V at a scan rate of 0.1 V s^−1^. The ECSAs determined from the H atom adsorption area are 8.92, 8.43, and 8.04 m^2^ g_(Pt)_^−1^ for the NPs prepared with the three different molar ratios of Au to Pt (3:7, 5:5, and 7:3), respectively (Fig. 4b). The ECSAs from cathodic peaks of Au were 0.05, 0.11, and 0.37 m^2^ g_(Au)_^−1^, respectively (Fig. 4c). As the proportion of Au increases, the ECSA from the adsorption of H atoms decreases (Fig. S4a), while the ECSA from cathodic peaks of Au increases (Fig. S4b). Furthermore, linear sweep voltammograms (LSVs) were recorded in 0.1 M KOH (pH 13.0) and PBS (pH 7.4) solution containing 10 mM glucose, respectively (Fig. S5). The oxidation peak currents in KOH solution (pH 13.0) for Au@Pt and Pt NPs were 30.3 and 26.77 μA, and the peak currents in PBS solution (pH 7.4) for Au@Pt and Pt NPs were 12.8 and 5.3 μA. Interestingly, Au NPs showed the very low oxidation current in the both solutions. As can be shown in Fig. S5, our Au@Pt NPs show the highest response current for the catalytic oxidation of glucose in pH 7.4 solution compared with those of Pt and Au NPs.

**Figure 5:**
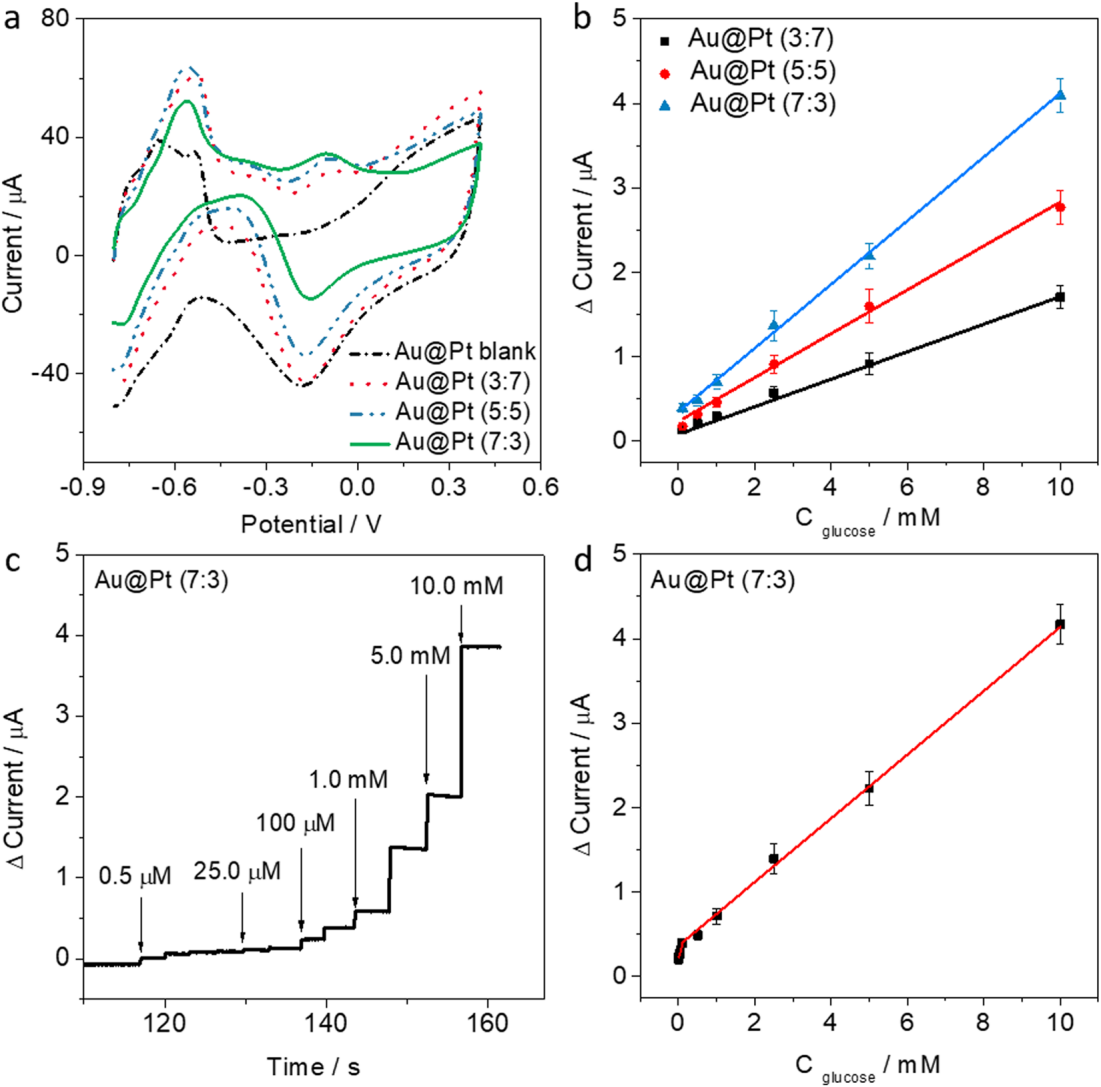
(**a**) CVs for Au@Pt NPs with three different Au:Pt ratios (3:7, 5:5, 7:3) recorded in 0.1 M PBS (pH 7.4) containing 10 mM glucose at a scan rate of 100 mV s^−1^. (**b**) Calibration plots of modified electrodes: Au@Pt (3:7) (black line), Au@Pt (5:5) (red line), and Au@Pt (7:3) (blue line). (**c**) Amperometric response of Au@Pt (7:3) modified electrode to a wide range of glucose concentrations in 0.1 M PBS (pH 7.4), and (**d**) calibration plot of Au@Pt (7:3) modified electrode (applied potential: +0.10 V (Ag/AgCl)).

To determine the best content ratios of Au to Pt (3:7, 5:5, and 7:3), firstly, we optimized the Au@Pt drop-coating amount on the SPCE and the applied potential for electrochemical experiments (Fig. S6). CVs were recorded in 0.1 M PBS solution containing 10 mM glucose (pH 7.4) (Fig. 5a). During the anodic scan from −0.8 V to +0.4, the glucose oxidation peak was observed at −0.09 V. The peak currents for each ratio (3:7, 5:5, and 7:3) were 3.28, 4.74, and 5.84 μA, respectively. As the content of Au increased, the current for the glucose oxidation peak also increased. In addition, the calibration plots obtained for the different glucose concentrations (from 0.1 to 100 mM) indicated that the sensitivity of the Au@Pt (7:3) NPs (0.3765 μA μM^−1^) was 2.3 and 1.7 times higher than for the Au@Pt (3:7 and 5:5) NPs (0.2823 μA μM^−1^ and 0.1615 μA μM^−1^), respectively (Fig. 5b). As a result, we selected the Au@Pt (7:3) NPs for further study, because Au@Pt (7:3) was the best for the glucose oxidation reaction compared to other ratios. When the proportion of gold is higher than that of platinum, it is clear that the glucose oxidation reaction is also facilitated. The chronoamperometric (CA) response of Au@Pt (7:3) NPs were observed in the range of glucose from 0.5 μM to 10.0 mM (Fig. 5c). As the concentration of glucose increased, the peak current for the glucose oxidation also increased. Two dynamic ranges of the calibration plot were observed for the glucose detection, which are from 0.5–50.0 µM and 0.05–10.0 mM, with correlation coefficients of 0.9963 and 0.9788, respectively (Fig. 5d). The detection limit (DL) of glucose was determined to be 319.8 (±5.4) nM. Table 1 shows a comparison of our performance with the previous literature^33–36^. Therefore, Au@Pt (7:3) NPs can be highly useful as an electrode material for glucose detection.

**Table 1:** Comparison of the performance of our Au@Pt NPs with previous reports.

Materials	Methods	Detection Limit	Dynamic ranges	Reference
Core-shell structured Au@Pt	CA	0.319 μM	0.5–50.0 µM and 0.05–10.0 mM	Our work
Nanoporous PtAu alloy	CA	~0.5 μM	Up to 5.4 mM	^33^
Mesoporous Pt-Au alloy films	CA	6.0 μM	6.0 μM –11 mM	^34^
Bimetallic Pt-Au	CA	7.7 μM	0.01–7.5 mM	^35^
PtAu/C	CA	2.0 μM	1–10 mM	^36^

## Conclusion

We have successfully synthesised Au@Pt nanoparticles (NPs) in different content ratios by the sonochemical method. Observations of the prepared Au@Pt NPs indicated that the Au is located in core, while the Pt is located in the outer shell. Interestingly, the thicknesses of the Pt shell regions were only changed by different content ratios. Based on XRD and TEM, these NPs display the metallic *fcc* structure. As the content of Pt decreased, the ECSA of Au increased, because the thickness of the Pt shell decreased. With these samples, we demonstrated catalytic glucose oxidation using Au@Pt (3:7, 5:5, and 7:3 ratios) in neutral solution (pH 7.4). We observed that the Au@Pt (7:3) NPs revealed the best performance compared to the other ratios. The calibration curve using Au@Pt (7:3) NPs clearly shows two dynamic ranges for glucose concentrations ranging from 0.5–50.0 µM and 0.05–10.0 mM, with the detection limit of 319.8 (±5.4) nM in neutral solution (pH 7.4). As a result, the Au@Pt (7:3) NPs can be used as the efficient glucose oxidation catalyst in neutral solution (pH 7.4), paving the way for practical application.

## Experimental

### Reagents and materials

Polyethylene glycol hexadecyl ether (Brij 58^®^, HO(CH_2_CH_2_O)_20_C_16_H_33_), potassium tetrachloroplatinate(II) (K_2_PtCl_4_, 98%), D-(+)-glucose, and gold(III) chloride trihydrate (HAuCl_4_·3H_2_O, ≥99.9% trace metals basis) were obtained from Sigma-Aldrich Co. (USA). A phosphate buffer solution (PBS) was prepared using 0.1 M sodium dihydrogen phosphate (NaH_2_PO_4_, Sigma-Aldrich Co. USA) and 0.1 M disodium hydrogen phosphate (Na_2_H_2_PO_4_, Sigma-Aldrich Co. USA). All aqueous solutions were prepared in doubly distilled water (18 MΩ cm).

### Preparation of different molar ratios of core-shell Au@Pt NPs

Three different ratios (3:7, 5:5, 7:3) of Au (HAuCl_4_, 20 mM) to Pt (K_2_PtCl_4_, 20 mM) in mixed solutions were prepared (5 ml). The solution was then mixed with Brij 58^®^ (0.05 g) in a vial, and 5.0 mL of ascorbic acid (AA) solution (0.1 M) was added. This solution was reacted at room temperature for 1 hour under ultrasonication (220–240 V, 50–60 Hz). The precipitates were collected by centrifugation and washed several times with a deionized (DI) water and ethanol mixed solution to remove the surfactant and excess reactants.

### Instruments

The morphology and structure of the different molar ratios (3:7, 5:5, 7:3) of Au@Pt were observed using TEM (JEOL-2010 and JEM-2100F, Japan) and SEM (JEOL 7500, JAPAN). Wide-angle powder XRD patterns were obtained with a GBC MMA XRD at a scan rate of 2° min^−1^. Size distribution analysis was obtained using SZ-100 (HORIBA, JAPAN). The screen-printed carbon electrodes (SPCEs) were composed of carbon, Ag/AgCl, and carbon as a three electrode system (working, reference, and counter electrodes). A solid-state Ag/AgCl reference electrode was prepared according to a previous report. The electrode was printed on a polystyrene-base film with carbon and silver inks (Jujo Chemical, Japan) employing a screen printer (Bando Industrial, Korea)^4^. Amperograms and cyclic voltammograms (CVs) were recorded using a potentiostat/galvanostat (Kosentech Model PT-1 and EG & G PAR Model PAR 273 A).

## Electronic supplementary material

Supplementary Information
