# Supplementary material for: Rationally designed bimetallic Au@Pt nanoparticles for glucose oxidation
Source: Sci Rep. 2019 Jan 29;9:894. doi: 10.1038/s41598-018-36759-5 (PMC6351680; doi:10.1038/s41598-018-36759-5)
Supplement: Supplementary file 1 — Supplementary Information [file 41598_2018_36759_MOESM1_ESM.docx]

**Rationally designed bimetallic Au@Pt nanoparticles for glucose oxidation**

Kyubin Shim^a^, Won-Chul Lee^b^, Yoon-Uk Heo^c^, Mohammed Shahabuddin^d^, Min-Sik Park^e,*^, Md Shahriar A. Hossain^f^, Jung Ho Kim^a,e,*^

^a^ Institute for Superconducting and Electronic Materials (ISEM), Australian Institute for Innovative Materials (AIIM), University of Wollongong, North Wollongong, NSW 2500, Australia.

^b^ Department of Chemistry and Institute of BioPhysio Sensor Technology (IBST), Pusan National University, Busan 46241, Republic of Korea.

^c^ Graduate Institute of Ferrous Technology (GIFT), Pohang University of Science and Technology (POSTECH), San 31, Hyoja-Dong, Pohang 37673, Republic of Korea

^d^ Department of Physics and Astronomy, College of Science, King Saud University, P.O. Box 2455, Riyadh 11451, Saudi Arabia.

^e^ Department of Advanced Materials Engineering for Information and Electronics, Kyung Hee University, 1732 Deogyeong-daero, Giheung-gu, Yongin-si, Gyeonggi-do, 17104, Republic of Korea.

^f^ School of Mechanical & Mining Engineering, The University of Queensland, Brisbane, QLD 4072, Australia.

*Corresponding authors:

J.H. Kim ([jhk@uow.edu.au](mailto:jhk@uow.edu.au)) & M.-S. Park ([mspark@khu.ac.kr](mailto:mspark@khu.ac.kr))


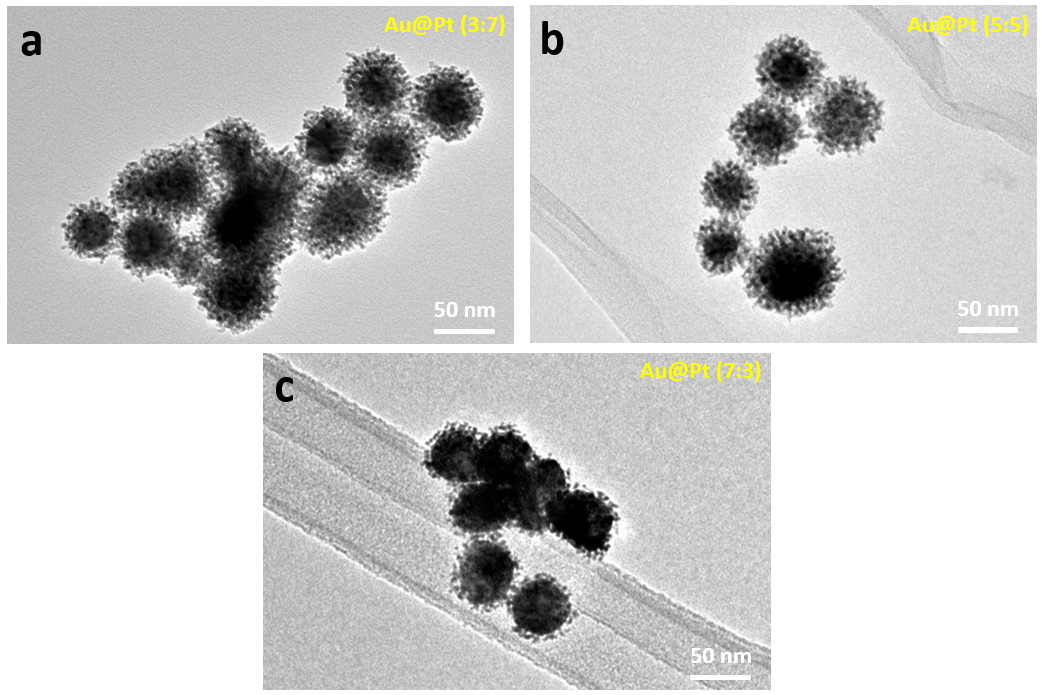


**Figure S1.** TEM images of different ration of Au@Pt NPs (a) 3:7, (b) 5:5, and (c) 7:3.


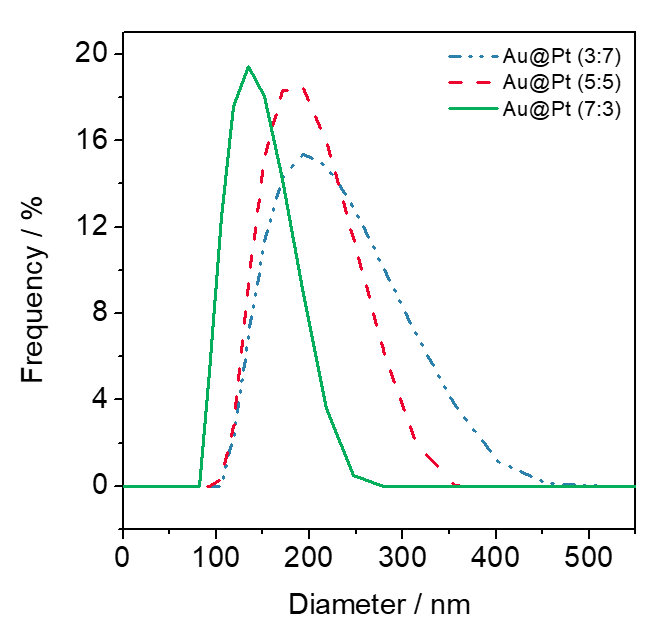


**Figure S2.** Particles size distribution analysis of Au@Pt NPs with three different Au:Pt Ratios (3:7 (blue, dash-dot-dot), 5:5 (red, dash), and 7:3 (green, solid)) by dynamic light scattering (DLS).


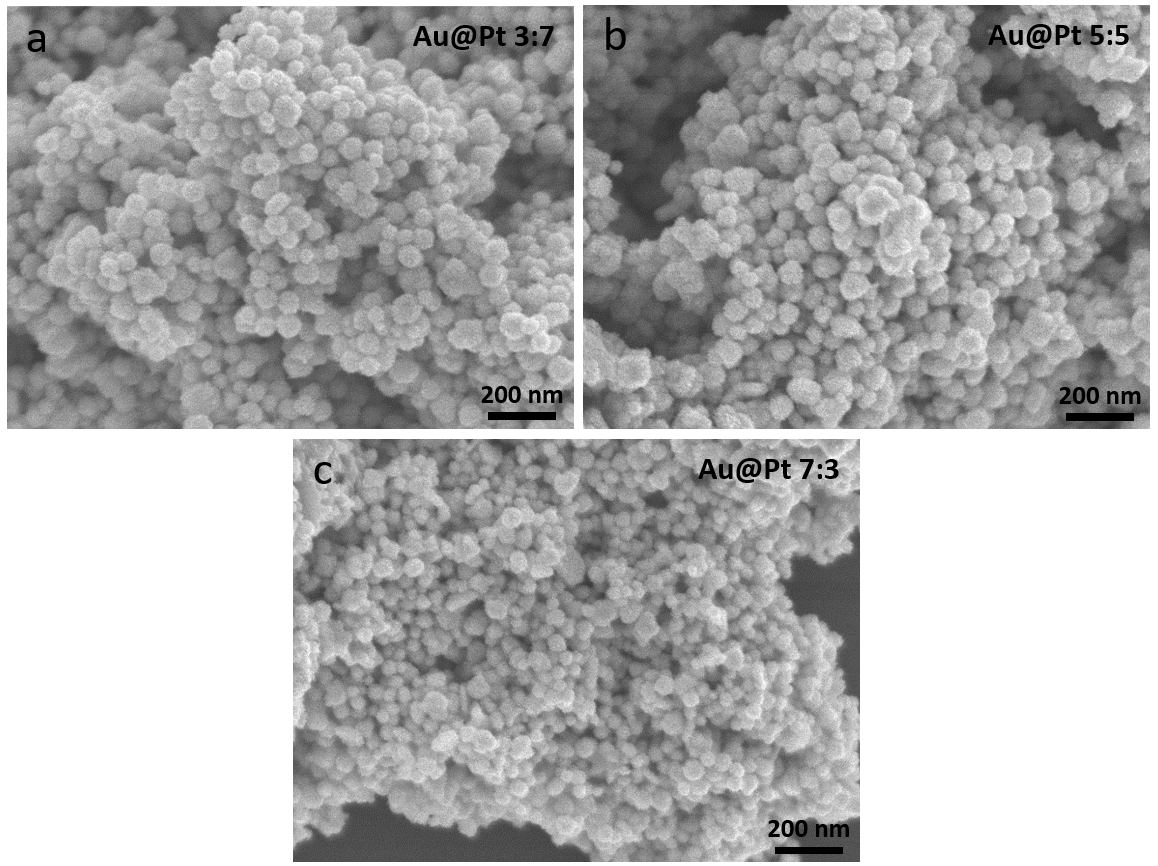


**Figure S3.** SEM images of Au@Pt NPs with three different Au:Pt ratios (a) 3:7, (b) 5:5, and (c) 7:3.


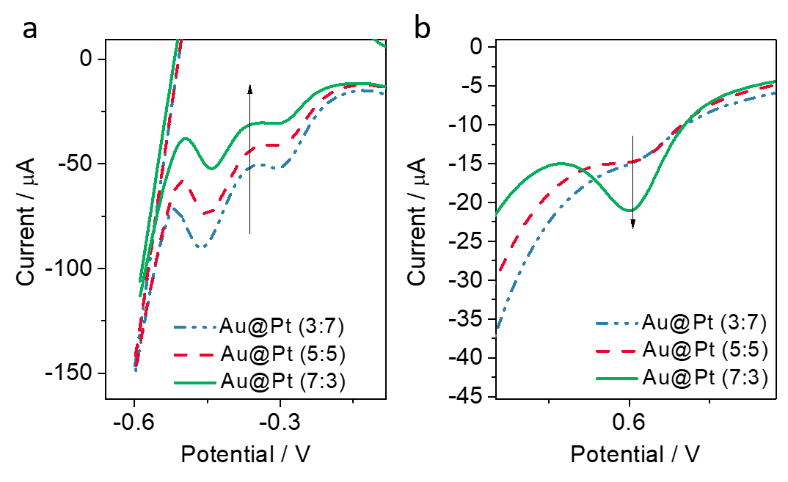


**Figure S4.** CVs of Au@Pt NPs with three different Au:Pt ratios (3:7, 5:5, 7:3): (a) adsorption of H atoms and (b) cathodic peaks of Au.


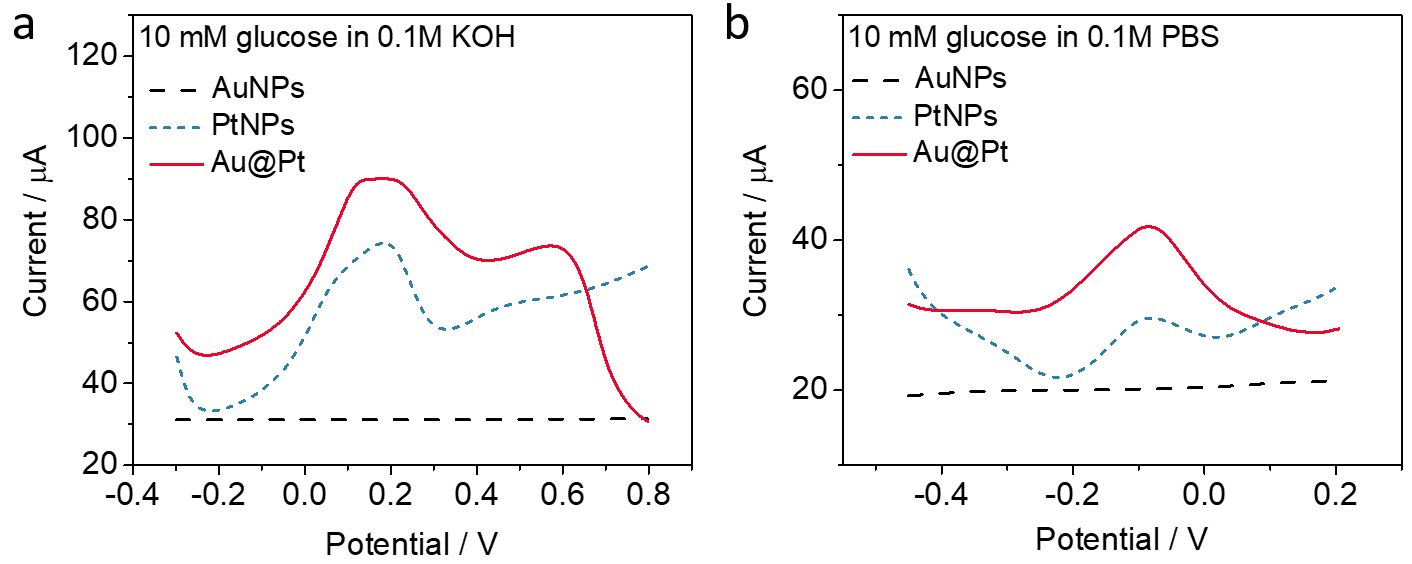


**Figure S5.** LSVs recorded for the electrodes: AuNPs/SPCE (dash line), PtNPs/AuNPs/SPCE (short dotted line), and Au@Pt/AuNPs/SPCE (solid line) in (a) 0.1 M KOH (pH 13) and (b) 0.1 M PBS (pH 7.4) containing 10 mM glucose, respectively.

**
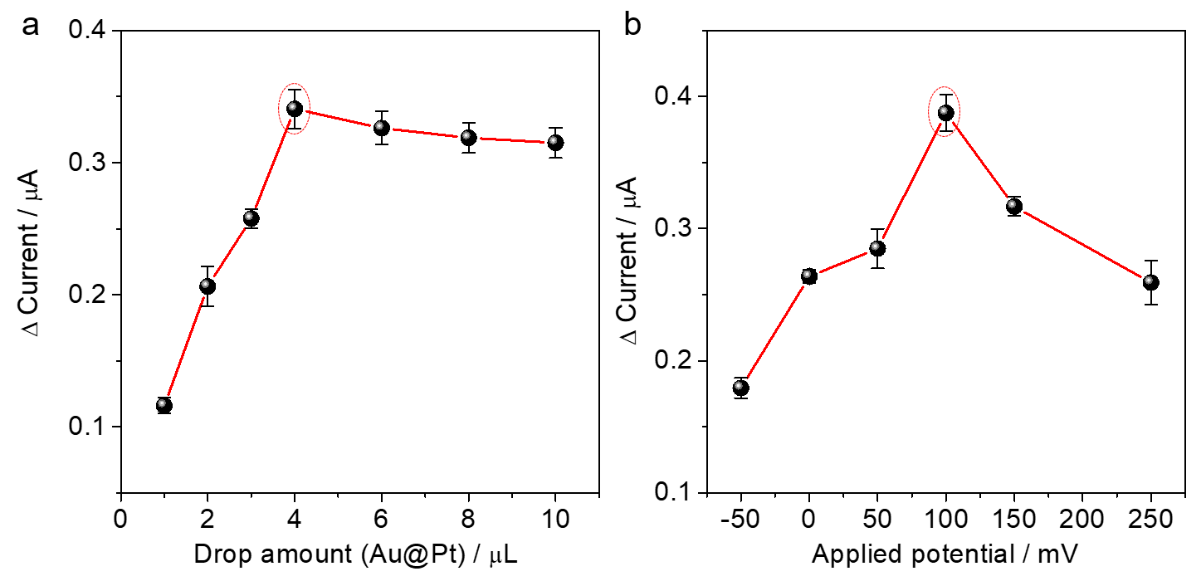
**

**Figure S6.** Optimization of Au@Pt NPs: (a) loading amount and (b) applied potential.
